# Acute physiological responses of blood flow restriction between high‐intensity interval repetitions in trained cyclists

**DOI:** 10.1002/ejsc.12107

**Published:** 2024-04-11

**Authors:** Charles F. Pugh, Carl D. Paton, Richard A. Ferguson, Matthew W. Driller, C. Martyn Beaven

**Affiliations:** ^1^ Te Huataki Waiora School of Health University of Waikato Hamilton New Zealand; ^2^ School of Health and Sport Science Te Pukenga The Eastern Institute of Technology Napier New Zealand; ^3^ School of Sport, Exercise and Health Sciences Loughborough University Loughborough UK; ^4^ Sport, Performance and Nutrition Research Group School of Allied Health, Human Services and Sport La Trobe University Melbourne Victoria Australia

**Keywords:** cardiorespiratory, physiology, recovery, stress, training

## Abstract

Blood flow restriction (BFR) is increasingly being used to enhance aerobic performance in endurance athletes. This study examined physiological responses to BFR applied in recovery phases within a high‐intensity interval training (HIIT) session in trained cyclists. Eleven competitive road cyclists (mean ± SD, age: 28 ± 7 years, body mass: 69 ± 6 kg, peak oxygen uptake: 65 ± 9 mL · kg^−1^ · min^−1^) completed two randomised crossover conditions: HIIT with (BFR) and without (CON) BFR applied during recovery phases. HIIT consisted of six 30‐s cycling bouts at an intensity equivalent to 85% of maximal 30‐s power (523 ± 93 W), interspersed with 4.5‐min recovery. BFR (200 mmHg, 12 cm cuff width) was applied for 2‐min in the early recovery phase between each interval. Pulmonary gas exchange (V̇O_2_, V̇CO_2_, and V̇E), tissue oxygen saturation index (TSI), heart rate (HR), and serum vascular endothelial growth factor concentration (VEGF) were measured. Compared to CON, BFR increased V̇CO_2_ and V̇E during work bouts (both *p* < 0.05, *dz* < 0.5), but there was no effect on V̇O_2_, TSI, or HR (*p* > 0.05). In early recovery, BFR decreased TSI, V̇O_2_, V̇CO_2_, and V̇E (all *p* < 0.05, *dz* > 0.8) versus CON, with no change in HR (*p* > 0.05). In late recovery, when BFR was released, V̇O_2_, V̇CO_2_, V̇E, and HR increased, but TSI decreased versus CON (all *p* < 0.05, *dz* > 0.8). There was a greater increase in VEGF at 3‐h post‐exercise in BFR compared to CON (*p* < 0.05, *dz* > 0.8). Incorporating BFR into HIIT recovery phases altered physiological responses compared to exercise alone.

## Highlights

- Blood flow restriction (BFR) during high‐intensity interval training recovery phases increased acute physiological perturbations and angiogenic markers compared to interval training alone.
- During the early recovery phases of high‐intensity interval cycling, BFR decreased muscle oxygenation, V̇O_2_, V̇CO_2_, and V̇E compared to CON condition with unrestricted recovery.

## 1 INTRODUCTION

The volume, intensity, and frequency of exercise performed during a training program are key determinants of the degree of physiological and performance adaptation (Galán‐Rioja et al., 2023). Incorporating diverse training modalities is especially important in endurance sports like competitive cycling (van Erp et al., 2021) which require athletes to sustain efforts across maximal and sub‐maximal intensities. These demands necessitate training oxidative energy pathways for endurance performance as well as glycolytic energy pathways to support high‐intensity efforts (Jeukendrup et al., 2000). Low‐to moderate‐intensity training induces adaptations including increased stroke volume, capillary and mitochondrial density, and a shift toward type I muscle fibres (Furrer et al., 2023), thereby improving fatigue resistance (Maunder et al., 2021) and maximal oxygen uptake (V̇O_2max_) (Ferretti et al., 2022). However, high‐intensity interval training (HIIT) provides additional benefits like enhanced buffering and oxygen‐independent energy metabolism (Gibala et al., 2009; Seiler & Tønnessen, 2009) that further augments intense endurance performance. Consequently, integrating short‐duration, high‐intensity intervals into training regimens prior to competition is an established strategy for endurance athletes (Laursen & Jenkins, 2002). For example, team‐pursuit track cyclists complete large volumes of low‐to moderate‐intensity training before incorporating high‐intensity practice of competition demands (Olaf Schumacher & Mueller, 2002). This sequenced approach may develop the superior critical power and rate of recovery measured in elite team‐pursuit cyclists (Pugh et al., 2022).

However, performance adaptations from HIIT can reach a plateau after 8–12 sessions (Paton & Hopkins, 2005) or ∼3‐week of training (Norrbom et al., 2022) without progressive overload of intensity or duration. This plateau may be potentially attributed to a decline in the activity of transcription factors hypoxia‐inducible factor 1‐alpha (HIF1‐α) and peroxisome proliferator‐activated receptor gamma coactivator 1‐alpha (PGC‐1α) (Norrbom et al., 2022; Perry et al., 2010), both of which are key activators of vascular endothelial growth factor (VEGF), a principal driver of angiogenesis (Chinsomboon et al., 2009; Lee et al., 2004; Olfert et al., 2010). Therefore, novel training approaches may be used to provide additional stimulus to augment the adaptive response to HIIT in trained endurance cyclists.

Integrating blood flow restriction (BFR) into HIIT sessions may help overcome plateaus in performance progression by inducing an augmented physiological stress to promote adaptation without increasing mechanical workload (Ross et al., 2023). The use of BFR involves applying pneumatic cuffs or tourniquets to limbs to limit arterial blood inflow and occlude venous return. This altered blood flow profile facilitates physiological stressors such as reduced muscle oxygenation (McManus et al., 2018), enhanced vascular shear stress (Hudlicka & Brown, 2009; Preobrazenski et al., 2020), metabolite accumulation (Loenneke et al., 2011; Sakamaki‐Sunaga et al., 2012), and increased oxidative stress (Christiansen et al., 2018). The intensified physiological and metabolic stress associated with BFR exercise stimulates adaptive responses in skeletal muscle and the microvasculature (Ferguson et al., 2021). For example, greater upregulation of skeletal muscle PGC‐1α mRNA was observed following gravity‐induced BFR cycling performed at an intensity just below the onset of blood lactate accumulation (OBLA) compared to work‐matched exercise without BFR (Preobrazenski et al., 2020). Similarly, Larkin et al. (2012) showed low‐load knee extension exercise (40% 1 repetition maximum, 1RM) with BFR (220 mmHg) increased VEGF and HIF‐1*α* mRNA 4‐h post‐exercise compared to the same exercise without BFR.

The effects of BFR are influenced by exercise intensity and whether BFR is applied continuously or intermittently (Pignanelli et al., 2021). During low‐to moderate‐intensity exercise, BFR is often applied continuously to heighten metabolic perturbations. For example, continuous BFR (140–200 mmHg) during low‐intensity cycling (30% maximal aerobic power, MAP) increased V̇O_2max_, MAP, isometric strength, and power output at OBLA (de Oliveira et al., 2016). Additionally, continuous BFR (75–100 mmHg) during low‐load resistance training (20%–30% 1RM) enhanced skeletal muscle capillary density (Nielsen et al., 2020) and mitochondrial protein content (Groennebaek et al., 2018). However, during high‐intensity interval exercise, BFR may be applied intermittently during the interval recovery phases to minimise discomfort (Willis et al., 2018). Indeed, applying BFR during recovery phases between high‐intensity plantar flexion bouts enables high work rates and minimises discomfort compared to continuous BFR (Okita et al., 2019), though direct evidence during high intensity interval cycling remains to be determined.

Applying BFR intermittently during the recovery phase of high‐intensity exercise sustains intramuscular metabolite concentrations (Okita et al., 2019) and reduces muscle oxygenation (Ienaga et al., 2022; McManus et al., 2018; Mitchell, 2019; Solsona et al., 2021). Although a session of 4 × 30‐s sprints with BFR during recovery did not amplify the increase in PGC‐1*α* or VEGF mRNA expression compared to sprinting alone, there was an augmented HIF‐1*α* mRNA response with BFR (Taylor et al., 2016). A subsequent training study utilising a progressive number (4–7) of 30‐s sprint repetitions with intermittent BFR during recovery over 4‐week (Mitchell et al., 2019) suggested a trend toward enhanced angiogenesis signalling with chronic BFR application that may have reached statistical significance with a larger sample size. While BFR did not significantly enhance sprint training adaptation in trained individuals, it may provide added stimulus in trained athletes who have plateaued in their adaptation to traditional HIIT protocols. Therefore, the heightened physiological and metabolic stresses elicited by integrating BFR into the recovery phases of HIIT may potentiate physiological adaptations.

Few studies to date have investigated the magnitude of acute physiological perturbations experienced during the application of BFR in the recovery phases during HIIT. Therefore, the objective of this study is to investigate the acute physiological effects of a six‐repetition HIIT session either with BFR applied during the recovery phases or with an unrestricted recovery in trained cyclists. It was hypothesised that, compared to a standard HIIT session without BFR, applying BFR during the recovery phases would reduce cardio‐pulmonary and muscle O_2_ saturation measures, adding to the overall physiological stress of the training stimulus and increase serum VEGF concentration post‐exercise.

## 2 METHODS

### 2.1 Participants

Eleven competitive road cyclists (male *n* = 9, female *n* = 2) training for >6‐h · week^−1^ volunteered for the study (age: 28 ± 7 years; height: 175 ± 7 cm; body mass: 69 ± 6 kg; peak oxygen uptake, V̇O_2peak_: 4.5 ± 0.7 L · min^−1^, 65 ± 9 mL · kg^−1^ · min^−1^). Inclusion criteria for females required uninterrupted hormonal contraception use throughout the study to control for potential hormonal fluctuations. Participants' abilities were categorised as trained (tier three, *n* = 2), highly trained (tier four, *n* = 6) and professional (tier five, *n* = 3) based on established nomenclature (De Pauw et al., 2013). Prior to testing, participants completed a medical screening questionnaire to ensure they had no cardiovascular or haematological contraindications to BFR. Participants were screened prior to recruitment (Kacin et al., 2015) and provided written informed consent. The study was approved by the participating institution's human research ethics committee (ethics approval number: HREC(Health)2021#22) and performed in accordance with the Declaration of Helsinki.

### 2.2 Experimental design

This study employed a randomised crossover design with two conditions: HIIT with (BFR) or without (CON) BFR applied during recovery phases. Participants attended the laboratory on three occasions over a ∼21‐day period. During the first visit, participants completed an incremental ramp test to determine their V̇O_2peak_ and a maximal 30‐s sprint test. Participants also completed a partial familiarisation to the experimental protocol, which included two repetitions of the HIIT protocol with NIRS and BFR but without cardiopulmonary or blood sampling. In visits two and three, participants performed an individualised HIIT protocol consisting of six, 30‐s work bouts with either the experimental (BFR) or control (CON) condition applied during the recovery between work bouts.

All trials took place in an environmentally controlled laboratory (temperature 19 ± 1ºC; relative humidity 45 ± 5%). Experimental sessions were conducted at the same time of day (±1‐h) for each participant to control for diurnal variation and were separated by 3–7‐days. Participants performed all trials on their personal bicycles mounted to a stationary ergometer (Kickr V5, Wahoo), with resistance controlled via software (Trainer Road). Power output in watts (W) was recorded at 1 Hz by the cycle ergometer.

Participants were instructed to avoid strenuous physical activity and replicate any light training performed in the 24‐h prior to each testing session. Participants recorded their dietary intake for the 24‐h preceding the first experimental trial and replicated this as closely as possible for each subsequent trial. Participants abstained from caffeine‐containing products 12‐h before the tests and refrained from eating, consuming only water for the 3‐h before each testing session.

### 2.3 Preliminary testing procedures

#### 2.3.1 Incremental ramp test

Participants completed an incremental ramp exercise protocol while seated on the ergometer, starting at 150 W and increasing at 25 W · min^−1^ (∼0.4 W · s^−1^) (Racinais et al., 2014). Participants self‐selected a pedalling cadence between 75 and 100 revolutions per minute (RPM) and maintained this cadence throughout the duration of the test. Exercise continued until volitional exhaustion or when cadence fell 10% below the chosen rate for more than 5‐s, despite strong verbal encouragement. Pulmonary gas exchange was measured continuously throughout exercise with a metabolic cart (Parvo metabolic cart, Medics TrueOne 2400), which was calibrated according to the manufacturer's instructions. This experimental set‐up allowed for breath‐by‐breath analysis of oxygen (O_2_) uptake (V̇O_2_), carbon dioxide production (V̇CO_2_) and minute ventilation (V̇E). The highest mean V̇O_2_ measured over any continuous 30‐s period during the ramp test was defined as V̇O_2peak_. Maximal aerobic power (MAP) was defined as the mean power output during the final 5‐s of the test (Buchfuhrer et al., 1983). The mean MAP achieved by participants in the study was 409 ± 58 W and 5.91 ± 0.77 W · kg^−1^ in relative terms.

#### 2.3.2 Maximal 30‐s test

Following a 10‐min active‐recovery period after the incremental ramp test, participants completed a 10‐s familiarisation effort of the 30‐s maximal sprint test to establish appropriate gearing for a cadence of 80–120 RPM. The incremental test and 10‐s familiarisation functioned as a warm‐up for the 30‐s test. The maximal 30‐s test was performed while seated with the ergometer set in an isoinertial mode, 5‐min after the familiarisation. The procedure required participants to pedal at a constant cadence of 110 RPM before ergometer resistance was applied. Participants were provided strong verbal encouragement to produce a maximal effort throughout the 30‐s test. The test was deemed valid if the participant maintained their cadence within an 80–120 RPM range. The mean power output attained during the maximal 30‐s test defined the metric, P30s. Peak power output was recorded as the greatest 1‐s power output value. On average, participants attained a P30s of 625 ± 117 W and a peak power output of 894 ± 250 W.

### 2.4 Main experimental protocols

Upon arrival at the laboratory, participants rested in a seated position for 10‐min while they were fitted with two near‐infrared spectroscopy (NIRS) sensors to measure tissue O_2_ saturation index (TSI; %), recorded at 1 Hz (Moxy 3, Fortiori Design LLC). The NIRS sensors were secured at the same anatomical location between sessions to the left and right vastus‐lateralis muscle bellies at 40% of the distance between the greater trochanter and the lateral epicondyle of the femur. The sensors were placed in a light shield and secured in place with a compression bandage. Heart rate (HR) was measured continuously via telemetry (H10, Polar Electro). A venous blood sample was obtained 10‐min before the warm‐up. With the ergometer set in constant power mode, participants then performed a 20‐min individualised warm‐up which consisted of 5‐min at 35% of MAP, 3‐min at 50% of MAP, 1‐min at 60% of MAP, 1‐min at 75% of MAP and 10‐min at 30% of MAP.

Experimental HIIT exercise trials began immediately after the warm‐up following a 5‐s countdown. Pilot studies informed that team‐pursuit lead power output was approximately 85% of an individual's mean power output during the maximal 30‐s test (85% of P30s). Therefore, the HIIT exercise trial involved six, 30‐s work bouts at 85% of P30s (523 ± 94 W), interspersed with ∼4.5‐min of passive recovery in a supine position on an adjacent bed (work‐to‐recovery ratio = 1:9). With 30‐s remaining before their next work bout, participants remounted their bicycles to continue the subsequent repetition. At this point, they could cycle freely with zero resistance applied to the ergometer and were instructed to increase their pedal cadence to 100–120 RPM just before the start of each work bout. Breath‐by‐breath pulmonary gas exchange was measured throughout the exercise session (analysis detailed below). After completing the exercise session, participants were allowed to leave the laboratory but required to fast (except drinking water) and avoid exercise, before returning to the laboratory 3‐h later to provide a post‐exercise venous blood sample.

#### 2.4.1 BFR protocol

In the BFR condition, once the participant lay semi‐supine, 12 cm wide occlusion cuffs (Occlude) were applied to the uppermost portion of both thighs. The cuffs were then inflated to 200 mmHg using hand‐operated sphygmomanometers, which was the highest tolerable pressure determined during pilot testing in a similar cohort. This pressure was chosen with the aim of achieving close to complete arterial occlusion in most participants, while avoiding intolerable discomfort. Although this standardised pressure likely surpassed 100% arterial occlusion pressure in some participants, determining individualised arterial occlusion pressure was not feasible due to lack of reliable equipment. Based on the thigh circumferences of our participants (56.7 ± 2.3 cm, measured at a proximal point one‐third of the distance between the inguinal fold and superior patella), we estimate 200 mmHg represented approximately 80%–120% of mean arterial occlusion pressure. For reference, Hunt et al. (2016) found ∼185 mmHg was the mean pressure necessary for poplitaeal artery occlusion using 13 cm width cuffs, while Loenneke et al. (2012) found 13.5 cm cuffs inflated to ∼156 mmHg was the mean cuff pressure for posterior tibial artery occlusion. The cuff application and inflation process took approximately 30‐s from the end of the work bouts. Pressure was maintained for 2‐min before deflation and cuff removal to provide participants with 1.5‐min of unoccluded supine recovery. The BFR and CON conditions were workload‐ and exercise‐duration matched.

#### 2.4.2 Blood sampling and analysis

Blood samples were taken by venepuncture (21G, Vacutainer, PrecisionGlide, BD) from antecubital veins with the participant seated. Blood samples for serum (SST II Advance Gold, 8.5 mL, Vacutainer, BD) were immediately inverted five times following collection, then rested at room temperature for 10‐min before being placed inside an ice‐cooled, insulated container. Blood samples were centrifuged within 2‐h of collection at 1500 G for 10‐min before serum was aliquoted and stored at −20°C. Serum samples were analysed, in triplicate, using human VEGF‐A ELISA plates (Invitrogen, Thermo Fisher Scientific) with automated plate washing and reading. All samples for a given individual were analysed on a single plate. The intra‐assay coefficient of variation of ELISA analyses was 5.1% and the inter‐assay CV was 6.7% and concentrations reported in pg · mL^−1^.

#### 2.4.3 Data handling

A cycling computer (Edge 520, Garmin) was used to capture the raw power output from the ergometer, NIRS devices, and HR monitor. The raw data file was downloaded to Golden Cheetah (version 3.5, open source, https://www.goldencheetah.org/) for subsequent analysis. These data were then imported into an Excel spreadsheet (Microsoft). Similarly, raw metabolic data were incorporated into the same spreadsheet and synchronised with the exercise onset using event markers from both the cycling computer and metabolic cart. Mean power output was calculated for each 30‐s work bout. Mean values for TSI, HR, V̇O_2_, V̇CO_2_, and V̇E were calculated over entire HIIT sessions and separately categorised into three phases: the work bouts (0–30‐s), the early recovery phase (occluded: 1–3‐min), and the late recovery phase (unoccluded: 3–4.5‐min). For analysis, mean values for TSI, V̇O_2_, V̇CO_2_, V̇E, and HR were calculated for each phase across all six repetitions and participants. Mean values for each variable and condition were also determined over entire recovery phases (combined early and late recovery phases). In addition, traces from all participants were averaged into 5‐s bins for each variable to create a single visual representation.

### 2.5 Statistical analysis

Separate one‐way repeated‐measures ANOVA were conducted to assess the effect of condition on entire recovery and whole sessions. A two‐way 2 × 6 repeated‐measures ANOVA was conducted with ‘condition’ (BFR and CON) and ‘repetition’ (1–6) (each divided into time phases of work bouts, early recovery, and late recovery) as factors, comparing power output, TSI, V̇O_2_, V̇CO_2_, V̇E, and HR means. The interaction effects between condition and repetition, as well as condition and HIIT phase, were examined. To determine the effect of BFR on angiogenic signalling, a two‐way repeated‐measures ANOVA was run on serum VEGF concentration with two within‐subject factors: ‘time’ (pre‐exercise, 3‐h post‐exercise) and ‘condition’ (BFR, CON). Where significant interaction effects were found, Bonferroni‐corrected *post‐hoc* analyses were performed. Residual normality was confirmed by the Shapiro‐Wilk test. Mauchly's test indicated that the assumption of sphericity was violated for the main effects of repetition (*p* < 0.05); therefore, degrees of freedom were corrected using Greenhouse–Geisser estimates. The statistical analyses were performed using SPSS (IBM, SPSS for Windows, Version 29.0), with statistical significance set at *p* ≤ 0.05. Graphs were produced in GraphPad Prism 9.5.1 for Windows (GraphPad Software). If an effect was significant, the magnitude of effect sizes were interpreted using *dz* thresholds of 0.2, 0.5 and 0.8 for *small*, *moderate* and *large* (Cohen, 1988). An effect size of *dz* < 0.2 was considered *trivial*. Data are presented as mean ± 95% confidence intervals constructed by the normal approximation method.

## 3 RESULTS

### 3.1 5‐s trace interpretation

**FIGURE 1:**
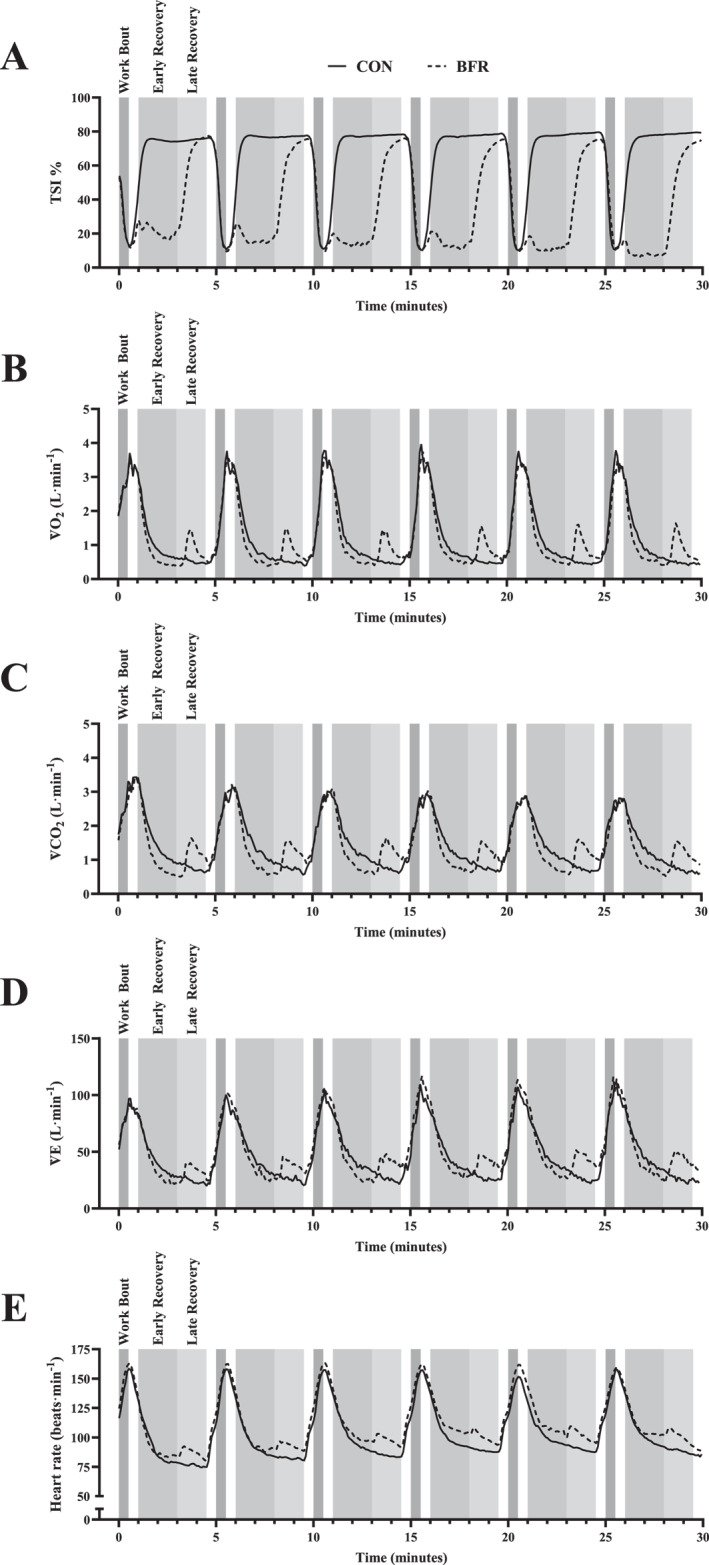
Mean 5‐s traces of physiological parameters for BFR and CON interventions. Phases are labelled in the first repetition, with consistent shading thereafter. Panels: (A) tissue saturation index (TSI); (B) oxygen uptake (V̇O_2_); (C) carbon dioxide production (V̇CO_2_); (D) minute ventilation (V̇E); and (E) heart rate (HR) for blood flow restriction (BFR) and control (CON) conditions.

Figure 1 shows 5‐s averaged traces representing the mean across all participants for each experimental condition and variable. During the early recovery phase of the BFR condition, there was a reduction in TSI, V̇O_2_, V̇CO_2_, and V̇E compared to CON. However, during the late recovery phase, BFR cuff release led to TSI returning towards CON levels, while V̇O_2_, V̇CO_2_, and V̇E rise above CON levels. The effect of BFR is maintained for TSI, V̇O_2_, and V̇CO_2_ across all repetitions. The BFR condition led to an elevated HR during recovery compared to CON and led to an increase in V̇E across repetitions.

### 3.2 Power output

There was no significant main effect of condition (BFR vs. CON) on power output (*p* = 0.597; mean ± SD: BFR 523 ± 94 W, CON 523 ± 92 W). Power output for BFR and CON, respectively, was: repetition 1, 521 ± 95 W and 523 ± 94 W; repetition 2, 526 ± 95 W and 524 ± 94 W; repetition 3, 525 ± 95 W and 524 ± 93 W; repetition 4, 527 ± 96 W and 524 ± 93 W; repetition 5, 526 ± 94 W and 523 ± 92 W; repetition 6, 516 ± 93 W and 517 ± 89 W. The condition by repetition interaction on power output was not statistically significant (*p* = 0.237). There was no main effect of repetition on power output (*p* = 0.630).

**FIGURE 2:**
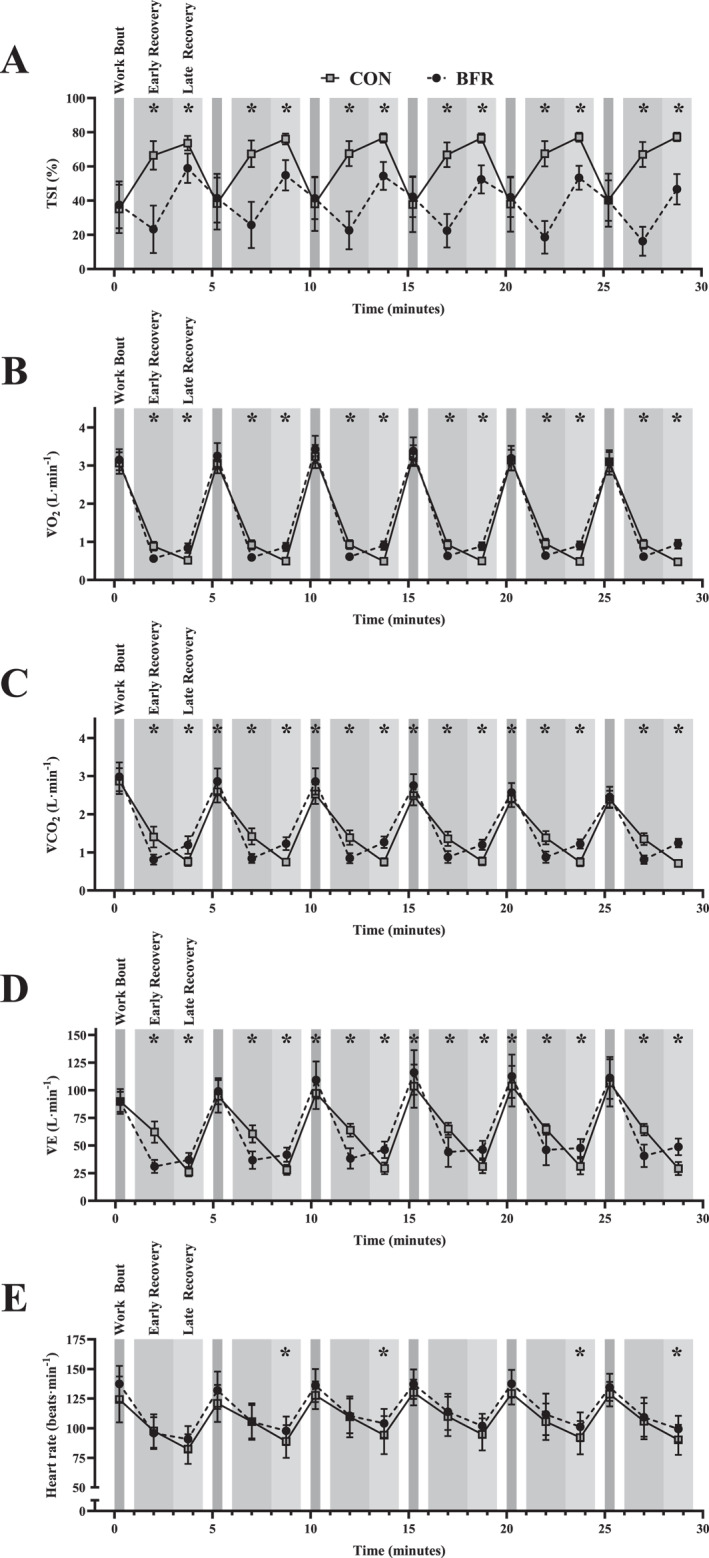
Physiological responses during BFR and CON interventions. Phases are labelled in the first repetition, with consistent shading thereafter. Panels: (A) tissue saturation index (TSI); (B) oxygen uptake (V̇O_2_); (C) carbon dioxide production (V̇CO_2_); (D) minute ventilation (V̇E); and (E) heart rate (HR) for blood flow restriction (BFR) and control (CON) conditions. Values are presented as mean ± 95% confidence intervals. An asterisk (*) indicates a significant (*p* < 0.05) post‐hoc result between conditions.

Figure 2 shows the mean data for all variables across all sets and phases from all participants. Table 1 shows the data for the measured physiological variables during the different phases of the HIIT session.

**TABLE 1:** Comparison of physiological parameters between BFR and CON interventions.

	Averaging period	Condition		Cohen's *dz* for condition means
Measure		BFR	CON	
TSI (%)	Entire session	35.6 ± 5.4^a^	64.0 ± 2.4	**2.58 ± 0.60**
	Entire recovery	35.2 ± 7.9^a^	71.0 ± 4.7	**2.00 ± 0.59**
	Work bout	37.5 ± 9.9	32.5 ± 14.1	0.26 ± 0.39
	Early recovery	21.5 ± 10.6^a^	67.1 ± 7.5	**6.23 ± 2.28**
	Late recovery	53.5 ± 7.5^a^	76.2 ± 2.9	**5.77 ± 2.58**
V̇O_2_ (L·min^−1^)	Entire session	1.21 ± 0.10	1.22 ± 0.10	0.05 ± 0.21
	Entire recovery	0.73 ± 0.07	0.74 ± 0.09	0.07 ± 0.28
	Work bout	3.35 ± 0.35	3.25 ± 0.30	0.24 ± 0.32
	Early recovery	0.61 ± 0.07^a^	0.92 ± 0.11	**2.11 ± 0.37**
	Late recovery	0.89 ± 0.11^a^	0.49 ± 0.06	**2.98 ± 0.39**
V̇CO_2_ (L·min^−1^)	Entire session	1.40 ± 0.14	1.43 ± 0.15	0.12 ± 0.20
	Entire recovery	1.01 ± 0.12^a^	1.11 ± 0.15	**0.47 ± 0.23**
	Work bout	2.61 ± 0.26^a^	2.80 ± 0.32	**0.44 ± 0.25**
	Early recovery	0.84 ± 0.12^a^	1.38 ± 0.19	**2.22 ± 0.28**
	Late recovery	1.23 ± 0.14^a^	0.74 ± 0.10	**2.38 ± 0.42**
V̇E (L·min^−1^)	Entire session	55.7 ± 9.1	51.9 ± 8.0	0.25 ± 0.26
	Entire recovery	41.7 ± 7.9^a^	48.7 ± 5.5	**0.60 ± 0.40**
	Work bout	109.0 ± 17.1^a^	101.7 ± 17.5	**0.29 ± 0.21**
	Early recovery	39.5 ± 9.1^a^	63.4 ± 6.2	**3.07 ± 1.15**
	Late recovery	44.6 ± 6.8^a^	29.1 ± 5.3	**1.50 ± 0.43**
HR (beats·min^−1^)	Entire session	114 ± 10^a^	106 ± 11	**0.48 ± 0.28**
	Entire recovery	104 ± 12	99 ± 14	0.29 ± 0.41
	Work bout	134 ± 12	127 ± 10	0.34 ± 0.60
	Early recovery	108 ± 14	106 ± 15	0.10 ± 0.32
	Late recovery	99 ± 10^a^	90 ± 13	**0.42 ± 0.34**

*Note*: Mean ± 95% CI of tissue saturation index (TSI), oxygen uptake (V̇O_2_), carbon dioxide output (V̇CO_2_), minute ventilation (V̇E) and heart rate (HR) at each time phase. Effect sizes for significant effects are presented in **bold.**

^a^ Indicate significant difference between blood flow restriction (BFR) and control (CON) conditions at that time point (*p* ≤ 0.05).

### 3.3 Tissue saturation index

Mean tissue saturation index (TSI) was significantly reduced with BFR compared to CON (*p* < 0.001), with a *large* (*dz* = 2.58) effect size. During work bouts, TSI did not differ between conditions (*p* = 0.496). However, TSI was significantly reduced with BFR during early (*p* < 0.001) and late (*p* < 0.001) recovery compared to CON, with *large* (*dz* = 6.23 and 5.77, respectively) effect sizes. There was a *large* (*dz* = 2.00) and significant reduction from BFR during the entire recovery TSI (*p* < 0.001), compared to CON. The interaction between condition and repetition on TSI was non‐significant (*p* = 0.093). There was no main effect of repetition on TSI (*p* = 0.396).

#### 3.3.1 V̇O_2_

V̇O_2_ during the work bout was not significantly affected by conditions (*p* = 0.193). However, BFR lead to a significant and *large* (*dz* = 2.11) reduction in early recovery V̇O_2_ (*p* < 0.001) and a significant and *large* (*dz* = 2.98) increase in late recovery V̇O_2_ (*p* < 0.001), compared to CON. Total recovery V̇O_2_ did not differ between conditions (*p* = 0.469). There was a significant main effect of repetition on V̇O_2_ (*p* = 0.040). *Post‐hoc* analysis demonstrated that the fourth repetition was greater than the second (*p* = 0.014). There was no significant interaction between condition and repetition on V̇O_2_ (*p* = 0.274). Mean session V̇O_2_ did not differ between BFR and CON (*p* = 0.540).

#### 3.3.2 V̇CO_2_

There was a *small* (*dz* = 0.44) but significant increase in work bout V̇CO_2_ with BFR compared to CON (*p* = 0.010). With BFR, there was a *large* (*dz* = 2.22), significant decrease in early recovery V̇CO_2_ (*p* < 0.001) and a *large* (*dz* = 2.38), significant increase in late recovery V̇CO_2_ (*p* < 0.001), compared to CON. There was a *small* (*dz* = 0.47) but significant reduction in entire recovery V̇CO_2_ with BFR (*p* = 0.001). There was a significant main effect of repetition on V̇CO_2_ (*p* = 0.013). *Post‐hoc* analysis demonstrated that V̇CO_2_ in the fifth repetition was less than the third (*p* = 0.018). There was no significant interaction between condition and repetition on V̇CO_2_ (*p* = 0.192). Mean session V̇CO_2_ did not differ between conditions (*p* = 0.478).

#### 3.3.3 V̇E

There was a *small* (*dz* = 0.29) but significant increase in work bout V̇E with BFR compared to CON (*p* = 0.044). Compared to CON, there was a significant and *large* (*dz* = 3.07) reduction in V̇E during early recovery with BFR (*p* < 0.001), whereas late recovery V̇E was significantly increased in BFR (*p* < 0.001) with a *large* (*dz* = 1.50) effect size. An interaction between condition and repetition (*p* = 0.012) indicated that with BFR, V̇E was greater in the third compared to first repetition (*p* = 0.044), and in the third compared to second repetition (*p* = 0.049). In CON, V̇E was greater in the fourth compared to second repetition (*p* = 0.009), and in the fifth compared to the second (*p* = 0.031). *Post‐hoc* tests for the simple main effect of condition at each repetition showed V̇E was greater in CON compared to BFR during the first repetition (*p* < 0.001). There was a significant main effect of repetition on V̇E (*p* = 0.004), with *post‐hoc* tests showing V̇E was greater in the third than the second repetition (*p* = 0.031). During the entire recovery, there was a significant and *moderate* (*dz* = 0.60) reduction in V̇E for BFR (*p* = 0.008), compared to CON. Mean session V̇E did not differ between conditions (*p* = 0.164).

#### 3.3.4 HR

There was no significant effect of condition on work bout HR (*p* = 0.127) or early recovery HR (*p* = 0.600). Compared to CON, BFR led to a *small* (*dz* = 0.42) but significant increase in HR during late recovery (*p* = 0.028). During the entire recovery, there were no significant differences in HR between conditions (*p* = 0.192). There was no significant interaction between condition and repetition on HR (*p* = 0.873). There was a *small* (*dz* = 0.48) but significant increase in mean session HR for BFR compared to CON (*p* = 0.017). There was no main effect of repetition on HR (*p* = 0.054).

#### 3.3.5 VEGF

**FIGURE 3:**
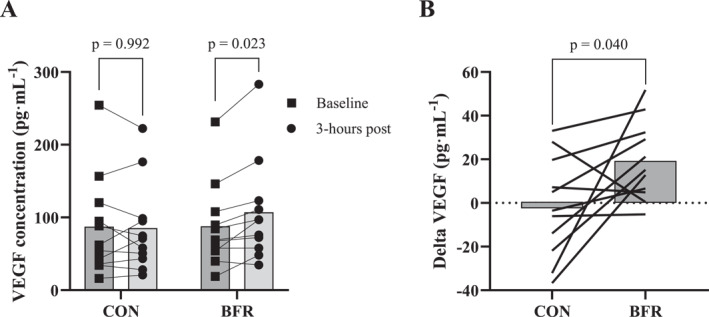
Acute vascular endothelial growth factor responses for BFR and CON. (A) Serum vascular endothelial growth factor (VEGF) at baseline and 3‐h post‐exercise for blood flow restriction (BFR) and control (CON) conditions. (B) Change in VEGF (post‐baseline) for BFR and CON. Bars are means. Brackets with *p*‐values show between‐condition comparisons at each timepoint in **A**. The *p*‐value in **B** indicates the condition × time interaction.

There was a significant interaction effect between condition and time on serum VEGF concentration (*p* = 0.040). There was a *small* (*dz* = 0.31 ± 0.18) but significant increase in serum VEGF concentration from baseline to 3‐h post‐HIIT in the BFR condition (Figure 3A). *Post‐hoc* analyses of change revealed a greater baseline to post‐intervention increase in the BFR condition (19 ± 11 pg · mL^−1^) compared to the CON condition (−1.9 ± 14 pg · mL^−1^) with a *large* effect size (*dz* = 0.91 ± 0.86) (Figure 3B).

## 4 DISCUSSION

Here we demonstrate that acute application of BFR during the early recovery phases of HIIT elicits significant reductions in TSI, V̇O_2_, V̇CO_2_, and V̇E during the BFR occlusion phase. Serum VEGF concentration was increased to a greater extent following HIIT with BFR compared to control. Upon reperfusion in the BFR condition during the late recovery phase, V̇O_2_, V̇CO_2_, V̇E, and HR rose beyond CON levels, while TSI remained reduced. The HIIT work bouts were unaffected by BFR in terms of power output, TSI, V̇O_2_, and HR. Although, BFR increased V̇CO_2_ and V̇E during the work bouts compared to CON. Over the entire HIIT protocol, BFR decreased TSI and increased HR, with no changes in V̇O_2_, V̇CO_2_, or V̇E. A progressive increase in V̇E with successive work bouts was observed with BFR. Collectively, these findings confirm our hypothesis by demonstrating BFR can acutely manipulate physiological responses when BFR is integrated into HIIT recovery phases.

In the early recovery phase, the substantial TSI reduction in the BFR condition aligns with previous studies applying BFR during recovery from high‐intensity exercise (Ienaga et al., 2022; McManus et al., 2018). The ∼68% decrease in TSI during the early recovery phase exceeded the ∼11% reduction reported by Mitchell (2019) despite the present study using a lower relative intensity, potentially reflecting Mitchell's lower occlusion pressure compared to the present study (∼125 vs. 200 mmHg, respectively). The BFR‐induced attenuation of TSI and V̇O_2_ imply constrained oxidative metabolism while diminished V̇CO_2_ implies reduced metabolite clearance from isolated peripheral circulation (Loenneke et al., 2010) and is consistent with the ‘metabolic freeze’ phenomenon (Okita et al., 2019). Further, the BFR‐induced reduction in TSI and V̇O_2_ following high‐intensity cycling suggests local hypoxia, which may stimulate the angiogenic response and is mediated, in part, by an increase in the activity of HIF1‐*α* (Rey & Semenza, 2010). Previous research shows that intramuscular stress elicited by moderate‐load resistance exercise with subsequent occlusion during recovery phases is caused by restricted phosphocreatine resynthesis and proton accumulation (Okita et al., 2019). These homoeostatic perturbations may activate the AMP‐activated protein kinase (AMPK), which in turn triggers the phosphorylation of PGC‐1*α* (Cantó et al., 2010), a key step in promoting mitochondrial biogenesis (Coffey & Hawley, 2007). Thus, rapidly inducing ischaemia via BFR after high‐intensity exercise restricts recovery processes and may augment both physiological and metabolic stress and downstream angiogenic and mitochondrial adaptive signalling. Herein, we provide evidence of an enhanced physiological stress in comparison to exercise alone.

The marked increases in V̇O_2_, V̇CO_2_, and V̇E during reperfusion in the BFR late recovery phase indicate re‐established peripheral and systemic circulation. The rapid TSI increase reflects restored O_2_ delivery, enabling oxidative phosphorylation and phosphocreatine resynthesis, as demonstrated by indices of muscle oxidative capacity when TSI is high (Pilotto et al., 2022). As phosphocreatine recovery kinetics align with V̇O_2_ after exercise (Hargreaves & Spriet, 2020), re‐phosphorylation likely contributes to the elevated V̇O_2_ in the present study. The elevated V̇CO_2_ during reperfusion reflects the requirement for bicarbonate buffering of hydrogen ions accumulated during ischaemia, as demonstrated previously (Okita et al., 2019). Therefore, the elevated V̇O_2_, V̇CO_2_, and V̇E following reperfusion facilitate normalisation of intramuscular pH while enabling metabolic recovery prior to subsequent work bouts. Repeated HIIT bouts with BFR can exacerbate metabolic accumulation and fatigue (McClean et al., 2023), evidenced here by a higher V̇CO_2_ and V̇E with BFR in subsequent bouts. However, there was evidence of greater physiological stress but not decreased exercise tolerance, as power output was similar between conditions through all repetitions.

The elevated serum VEGF concentration 3‐h following BFR provides putative evidence of an augmented angiogenic response (Hoier & Hellsten, 2014). While skeletal muscle cells contain vesicular stores and secrete substantial amounts of VEGF in response to muscle contractions (Høier et al., 2010), the significant increase in circulating VEGF following BFR is an encouraging finding. The elevated VEGF with BFR exercise may result from adaptive responses to reduced TSI caused by vascular occlusion as well as shear stress on the endothelium induced by compression of blood vessels (Hudlicka & Brown, 2009). Provided the acute elevation in serum VEGF also manifests as a chronic elevation with repeated BFR sessions, a sustained increase in circulating VEGF may induce long‐term adaptations in skeletal muscle O_2_ delivery and metabolite clearance (Tesch & Wright, 1983). Specifically, previous research has demonstrated that 3 weeks of daily resistance training (20% 1RM) performed to failure with concurrent BFR (100 mmHg) augments muscle O_2_ delivery and capillarisation compared to work‐matched unoccluded exercise (Nielsen et al., 2020). Furthermore, Mitchell et al. (2019) demonstrated that BFR (120 mmHg) applied during the recovery phases of a 4 weeks maximal sprint interval training intervention increased V̇O_2max_ to a greater extent than sprint interval training alone. The submaximal HIIT protocol used in the present study elicited an unchanged VEGF response in CON. Thus, BFR appears necessary to augment the VEGF response resulting from the current HIIT protocol in the present trained cyclist cohort.

A limitation of the present study is the 3‐h absence of participants from the laboratory before the post‐exercise blood sampling for VEGF. Additionally, the BFR cuff pressure was uniformly set at 200 mmHg rather than adjusted per individual using arterial occlusion pressure assessments, meaning that the degree of arterial occlusion varied between participants (Hunt et al., 2016; Loenneke et al., 2012). Furthermore, a single pair of BFR cuffs were used across all participants, leading to relatively different coverage of the upper thigh and overlap of cuffs between participants, which may have influenced the degree of occlusion (Bielitzki et al., 2021).

Our findings indicate that incorporating BFR into a HIIT regimen resulted in acute physiological changes while preserving a high‐intensity exercise stimulus. Importantly, participants could tolerate the BFR intervention and BFR did not impact the completion of their workouts. In fact, BFR occlusion only affected work bout V̇E and V̇CO_2_ with no impact on work bout power output, V̇O_2_, TSI, or HR. Our use of a standardised 200 mmHg BFR pressure allows for replication of this study in the field with minimal equipment, despite potential differences in the degree of occlusion between individuals. Coaches and athletes may consider integrating BFR into the recovery phases of HIIT to strategically modulate responses and induce targeted adaptations, depending on their goals and individual training programs. Given the endurance nature of intermittent high intensity cycling events (Pugh et al., 2022), the augmented physiological and metabolic stress responses from the present intervention may translate to additional muscular and cardiovascular adaptations with consistent training. Longitudinal intervention studies are necessary to substantiate the efficacy of the present study's intervention over a full training cycle to elucidate benefits for endurance athletes.

## 5 CONCLUSION

In conclusion, BFR during HIIT recovery intervals induced greater acute physiological perturbations compared to HIIT with unoccluded recovery in trained cyclists. Specifically, BFR reduced TSI, V̇O_2_, and V̇CO_2_ during the early recovery phase, while increasing V̇O_2_ and V̇CO_2_ in late recovery. There was also a greater concentration of serum VEGF 3‐h post HIIT with BFR compared to CON. These greater physiological stressors resulting from BFR have the potential to augment adaptive mechanisms that could benefit high‐intensity performance and endurance. Further research is warranted to investigate whether the acute effects of adding BFR to HIIT translate to measurable long‐term performance improvements in trained cyclists across a training period.

## CONFLICT OF INTEREST STATEMENT

The authors declare no potential conflict of interest.

## Data Availability

The data that support the findings of this study are available from the corresponding author upon reasonable request.
